# Metabolic engineering of endogenous MEP pathway for enhanced lycopene production in *Escherichia coli*

**DOI:** 10.1016/j.synbio.2026.01.014

**Published:** 2026-01-30

**Authors:** Xian Xu, Hongyu Xing, Hui Zhi, Chen Qin, Yuyue Deng, Wanqi Wei, Chunyan Huang

**Affiliations:** School of Food Science and Pharmaceutical Engineering, Nanjing Normal University, Nanjing, 210046, Jiangsu Province, China

**Keywords:** Lycopene, MEP pathway, *Escherichia coli*, Metabolic regulation, Gene source

## Abstract

Microbial cell factories represent the primary approach for heterologous lycopene synthesis, where gene source selection and pathway regulation have been demonstrated to have a significant impact on lycopene titer. In this study, key lycopene biosynthesis genes (*crtE*, *crtB* and *crtI*) derived from the extremophile *Deinococcus wulumuqiensis* R12 were introduced into *Escherichia coli*, generating the chassis strain H0. Fermentation optimization revealed sodium pyruvate significantly enhanced lycopene production and cell growth. Quantitative polymerase chain reaction (qPCR) analysis revealed that sodium pyruvate upregulated the expression of *dxr*, *ispA*, *crtE*, *crtB* and *crtI* genes, while downregulating the expression of *dxs* and *idi* genes. Consequently, different sources of *dxs*, *dxr*, *idi* and *ispA* genes were screened and co-expressed to reinforce the 2-C-methyl-d-erythritol 4-phosphate (MEP) pathway in *E. coli*. The optimized combination of *dxs* from *E. coli* MG1655 with *idi* from *D. wulumuqiensis* R12 achieved maximal lycopene titer of 293.70 mg/L (112.49 mg/g DCW), which was 33.88-fold higher than that of the initial strain H0. This study offers genetic resources for heterologous carotenoid synthesis and establishes a reference framework for the synthesis of analogous complex isoprenoid metabolites.

## 1 Introduction

**Fig. 1:**
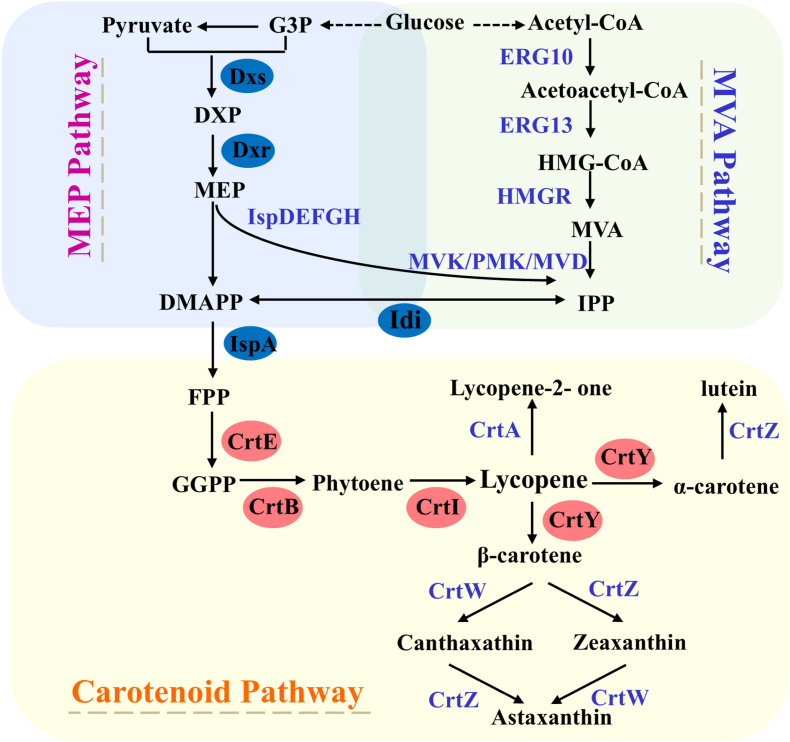
Biosynthetic pathways of carotenoids involving multiple enzymes. G3P: glyceraldehyde 3-phosphate; DXP: 1-deoxy-d-xylulose-5-phosphate; MEP: 2-C-methyl-d-erythritol-4-phosphate; DMAPP: dimethylallyl pyrophosphate; IPP: isopentenyl pyrophosphate; MVA: mevalonate; HMG-CoA: 3-hydroxy-3-methyl-glutaryl-CoA; GPP: geranyl pyrophosphate; FPP: farnesyl pyrophosphate; GGPP: geranylgeranyl pyrophosphate; DXS: 1-deoxy-d-xylulose-5-phosphate synthase; DXR: 1-deoxy-d-xylulose-5-phosphate reductoisomerase; IspD: 4-diphosphocytidyl-2-C-methyl-d-erythritol synthase; IspE: 4-diphosphocytidyl-2-C-methyl-d-erythritol kinase; IspF: 2-C-methyl-d-erythritol-2,4-cyclopyrophosphate synthase; IspG: 1-hydroxy-2-methyl-2-(E)-butenyl-4-pyrophosphate synthase; IspH: 1-hydroxy-2-methyl-2-(E)-butenyl-4-pyrophosphate reductase; IDI: isopentenyl pyrophosphate isomerase; IspA: farnesyl pyrophosphate synthase; ERG10: acetyl-CoA acetyltransferase; ERG13: hydroxymethylglutaryl-CoA synthase; HMGR: 3-hydroxy-3-methylglutaryl-coenzyme A reductate; MVK: mevalonate kinase; PMK: phosphomevalonate kinase; MVD: mevalonate 5-diphosphate decarboxylase; CrtE: GGPP synthase; CrtB: phytoene synthase; CrtI: phytoene desaturase; CrtY: lycopene cyclase; CrtZ: β-carotene hydroxylase; CrtW: β-carotene ketolase; CrtA: spheroidene monooxygenase.

As an important member of the isoprenoid family - lycopene is a highly lipophilic red carotenoid found in a variety of plants and microorganisms. Due to its unique physiological activities such as strong antioxidant and free radical scavenging to protect phagocytes from oxidative damage, it has been widely used in food, cosmetics, agriculture and other fields [1]. Lycopene can be produced not only by plant extraction or chemical synthesis, but also by biosynthesis by microorganisms. The biosynthetic process of lycopene is a multistep reactive metabolic pathway involving multiple enzymes and cofactors. The synthetic pathway uses isopentenyl pyrophosphate (IPP) and dimethylallyl pyrophosphate (DMAPP) as precursors. The main pathways for the synthesis of IPP and DMAPP in organisms are the 2-methyl-d-erythritol-4-phosphate (MEP) pathway in prokaryotes and plants and the mevalonate (MVA) pathway in eukaryotes [2]. The MEP pathway starts with pyruvate and glyceraldehyde 3-phosphate (G3P), and is catalyzed by 1-deoxy-d-xyloxy-xylulose-5-phosphate synthetase (encoded by *dxs*) and 1-deoxy-d-xyloxy-5-phosphate reductase (encoded by *dxr*) to form MEP, which is then catalyzed by five enzymes (encoded by *ispD*, *ispE*, *ispF*, *ispG*, and *ispH*, respectively) to form IPP and DMAPP. The MVA pathway begins with acetyl-coenzyme A, which is converted to 3-hydroxy-3-methylglutaryl monoacyl coenzyme A (HMG-CoA) by acetyl-coenzyme A acetyltransferase (encoded by *ERG10*) and hydroxymethylglutaryl coenzyme A synthase (encoded by *ERG13*). Then HMG-CoA undergoes 3 -hydroxy-3-methylglutaryl coenzyme A reductase (encoded by *tHMG1*) to generate MVA, and then IPP is generated by a three-step kinases catalyzed process. IPP and DMAPP can be interconverted by isopentenyl pyrophosphate isomerase (encoded by *idi*). IPP and DMAPP generated from these two pathways are catalyzed by farnesyl pyrophosphate synthase (encoded by *ispA*) to form farnesyl pyrophosphate (FPP), which enters the carotenoid pathway. FPP is then catalyzed sequentially by three key enzymes, geranylgeranyl pyrophosphate synthase (encoded by *crtE*), phytoene synthase (encoded by *crtB*) and phytoene desaturase (encoded by *crtI*) to form the first important carotenoid with color, lycopene, which undergoes chemical modification reactions such as cyclisation, oxygenation, dehydrogenation and acylation catalyzed by multienzymes, leading to the synthesis of other carotenoids and their derivatives of other structures [2,3](Fig. 1).

A variety of strains have been identified that can produce lycopene themselves, such as *Blakeslea trispora*, *Dietzia natronolimnaea*, *Rhodotorula glutinis*, *Haematococcus pluvialis* etc. [3]. However, the low yield of wild-type strains and insufficient supply of precursors limit the large-scale industrial production of lycopene. The combination of metabolic engineering and synthetic biology techniques can be used to mine key genes for lycopene synthesis from wild-type strains and transfer these genes into hosts for heterologous expression and regulation, e.g., *Escherichia coli*, yeast etc. *E*. *coli* is widely used as a primary model host for biosynthesis of terpenoid and high-value compounds due to its rapid growth rate, extensive physiological, biochemical and metabolic knowledge available as well as diverse engineering tools [4]. Current research strategies have focused on increasing the lycopene yield in *E. coli* by enhancing the MEP pathway of *E. coli* [[5], [6], [7], [8]], introducing exogenous MVA pathways [[9], [10], [11]], inhibiting or knocking out competing pathways [12], enhancing other metabolic pathways that provide precursors/intermediates/cofactors/energy [13], and enhancing the expression of multiple genes in the lycopene synthesis pathway [14,15]. Among these strategies, the use of expression of key genes in the MEP pathway, and the subsequent expression of three key enzymes, CrtE, CrtB and CrtI, is the major and easy-to-implement regulatory strategies to enhance the endogenous low MEP metabolic pathway in *E. coli* to meet substrate requirements. The two enzymes in MEP pathway, DXS and IspA from *Vibrio* sp. dhg were heterologously introduced in *E. coli* by Kim et al. leading significantly higher lycopene production compared to that of endogenous enzymes [16]. Hu et al. first integrated the exogenous *crtEBI* genes from *Corynebacterium glutamicum* 14067 into the chromosome of engineered *E. coli*, after knockdown modification and then overexpression of four genes (*dxs*, *dxr*, *ispA* and *idi*) involved in the MEP pathway, the highest lycopene yield was achieved at 25.82 mg/g, which showed that *E. coli* is an optimal chassis for ambitious metabolic modification to produce lycopene [17]. Heterogenous expression of *crtEBI* from *C*. *glutamicum* combined with overexpression of endogenous *dxs*, *dxr*, and *ispA* in 10 LPS mutant *E. coli* yielded a maximum lycopene yield of 5.39 mg/g DCW, representing a 142 % increase relative to the strain expressing *crtEBI* gene [18]. These examples demonstrate that the selection of different exogenous gene sources and the regulation of MEP pathway can significantly improve lycopene titer.

In this study, base on the previous optimization of the *crtE*, *crtB*, and *crtI* gene sequences derived from *Deinococcus wulumuqiensis* R12, which strengthened the lycopene pathway [14]. Fermentation was optimized to obtain strian H0 with high lycopene titer, in which the expression levels of genes related to the endogenous MEP pathway were regulated by overexpression. The two endogenous metabolic pathways (including the MEP pathway and the lycopene synthesis pathway) were regulated in strain H0 by increasing the copy number of key enzymes, up-regulating the expression of key genes in the MEP pathway, and screening *dxs*, *dxr*, *idi*, and *ispA* genes from different sources, and a platform strain with high lycopene titer was finally obtained. By constructing multi-gene expression plasmids in *E*. *coli*, the multienzyme synthetic pathway of carotenoids was established, and the lycopene titer was improved by fine regulation of metabolic engineering. The regulatory mechanism of key genes in the MEP pathway on lycopene synthesis was explored, and the synergistic effect between multienzymes were investigated, providing reference and demonstration for the synthesis of similar complex secondary metabolites.

## 2 Materials and methods

### 2.1 Strains, plasmids and culture media

*E. coli* BL21 (DE_3_) was used for the expression of the lycopene biosynthetic genes and lycopene fermentation. The plasmids and strains used in this study are listed in Table S1. 2 YT medium (yeast extract 10 g/L, tryptone 16 g/L, sodium chloride 5 g/L) was used as the seed medium and fermentation medium of genetically engineered bacteria. Recombinant *E. coli* was grown in 2 YT liquid medium at 37 °C, 200 rpm for 12 h and inoculated into 50 mL of fresh fermentation medium at a 2 % inoculum. The fermentation was induced by adding 4 g/L lactose after 2 h of incubation at 37 °C and 200 rpm in the dark, and the fermentation temperature was changed to 20 °C to start lycopene production. The recombinant strains were cultured in medium containing the appropriate antibiotics (50 μg/mL ampicillin or 25 μg/mL kanamycin sulfate). To determine the dry cell weight (DCW), 1 mL of the sample was centrifuged at 12,000×*g* for 5 min. The pellet was washed twice with deionized water, centrifuged again and dried at 100 °C until constant weight [14]. Dissolved oxygen (DO) in the liquid medium was measured by a portable dissolved oxygen meter equipped with a DO electrode (NineFocus NF4000, Mettler Toledo, Switzerland). The DO in shake-flasks was directly measured by the DO electrode inserted into the culture medium [19].

### 2.2 DNA manipulation and plasmid construction

The primers used for cloning are listed in Table S2. To optimize multigene co-expression, a medium-copy pETDuet-1 plasmid carrying the pBR322 origin of replication was employed. Genes *crtE*, *crtB* and *crtI* of *D. wulumuqiensis* R12 were obtained by PCR amplification from previously constructed plasmid pET-IE_1_B_1_ [20] and sequentially cloned downstream of two T7 promoters. The *crtEB* fragment was obtained by overlap PCR ligation of *crtE* and *crtB* genes, where *crtB* gene with ribosome binding site SD + AS sequence (TAAGGAGGATATACAT) [20]. The recombinant plasmid pET-IEB was obtained by double digestion of *crtI* ligated to the first multiple cloning site (MCS1) of pETDuet-1 plasmid and *crtEB* ligated to the second multiple cloning site (MCS2) (Fig. S1a). The recombinant plasmid pET-IEB was transformed into *E coli* BL21 (DE_3_) to obtain lycopene producing strain H0. In order to construct multi-copy recombinant plasmids and recombinant strains of CrtE, CrtB and CrtI, the genes *crtE*, *crtB* and *crtI* were cloned into the plasmid pRSFDuet-1 and then transferred into the chassis strain H0 to obtain the recombinant strains H0E, H0B, and H0I, respectively (Fig. S1b). Single-gene expression vectors and multigene co-expression vectors containing the *dxs*, *dxr*, *idi* and *ispA* genes of *E. coli* MG1655 and *R12dxs*, *R12dxr*, *R12idi* and *R12ispA* genes of *D. wulumuqiensis* R12 were transformed into strain H0 to obtain the corresponding dual-plasmid recombinant strains, respectively (Fig. S1c and d).

### 2.3 Extraction and detection of lycopene

The fermentation broth was centrifuged at 12,000 rpm for 1 min, and washed twice with ddH_2_O, the supernatant was discarded and 1 mL of acetone solution containing 1 g/L 2,6-di-tert-butyl-4-methylphenol (BHT) was added for lycopene extraction. The supernatant was obtained by centrifugation to determine the lycopene content using high-performance liquid chromatography (HPLC) [14]. The data represent the means ± standard deviations of three independent experiments.

### 2.4 Quantitative real-time PCR (qRT-PCR) analysis

Total RNA was isolated from samples using the EASYspinPLus Bacterial RNA Rapid Extraction Kit (Aidlab, China). The cDNA was synthesized using Hifair® II 1st Strand cDNA Synthesis SuperMix for qPCR (Yeasen Biotechnology, China). Quantitative real-time PCR amplification was carried out using the Hieff UNICON® qPCR SYBR Green Master Mix (Yeasen Biotechnology, China). All primers used in this study are listed in Table S2. The experimental design consisted of the group containing 25 g/L of sodium pyruvate and the control group devoid of sodium pyruvate supplementation. Bacterial cells were harvested by centrifugation (8000×*g*, 10 min, 4 °C) at 48 h after inoculation for qPCR. The *idnT* gene was used as the internal standard for relative quantification [21]. The relative quantitative analysis was evaluated according to the 2^−^^△△Ct^ method.

### 2.5 The stability of strain H21

Strain H21 was cultivated under optimal fermentation conditions, and its stability was measured as described previously [20]. The culture was subcultured in 2 YT medium every 48 h for 10 cycles to evaluate genetic stability. The culture was collected and diluted 10^4^-fold with 2 YT medium and then plated on 2 YT agar plates at 37 °C for 16 h with or without antibiotics to determine the numbers of colony-forming units (CFUs). Plasmid stability was calculated as the ratio of antibiotic-resistant CFUs to total CFUs.

### 2.6 Statistical analysis

Each set of experiments was conducted three times in parallel, and the error bars represent the standard deviation. Origin 2023 and SPSS 21.0 software with One-way ANOVA were used for significant difference analysis. The different treatment groups were compared by using the Tukey's test and the different lowercase letters indicate statistically significant difference (p < 0.05).

## 3 Results and discussion

### 3.1 Construction of a chassis strain for heterologous lycopene synthesis

To obtain strains with lycopene production capacity, the lycopene biosynthesis genes *crtE*, *crtB* and *crtI* derived from *D. wulumuqiensis* R12 were amplified and sequentially integrated into the plasmid pETDuet-1 to obtain the recombinant plasmid pET-IEB (Fig. S1a). The obtained recombinant plasmid pET-IEB was transformed into *E. coli* BL21 (DE_3_) to generate the engineered strain H0, which served as the chassis strain for lycopene synthesis. For initial lycopene production evaluation, strain H0 was cultured in 2 YT medium at 37 °C and 200 rpm with 2 % inoculum under dark conditions, and the products were assayed. The results showed that strain H0 had the ability to produce lycopene and achieved a maximum lycopene production of 8.67 mg/L (4.78 mg/g DCW) at 36 h (Fig. S2).

Subsequently, the effects of external culture conditions such as carbon source, nitrogen source, fermentation time, inducer concentration, and induction temperature on lycopene production were systematically investigated. As shown in Fig. S3a, supplementation with sodium pyruvate and glycerol in 2 YT medium increased the lycopene titer compared to the control. In addition, the addition of sodium pyruvate also significantly improved cellular biomass as evidenced by a nearly 2-fold increase in OD_600_ (11.94 vs. 6.05) relative to the control. In contrast, alternative carbon sources (glycerol, lactose, sucrose, glucose, fructose, maltose) failed to improve lycopene titers, with glucose and fructose exhibiting inhibitory effects. The effects of different concentrations of sodium pyruvate on lycopene synthesis were evaluated. The results showed that lycopene production increased with increasing concentration when the concentration of sodium pyruvate was less than 25 g/L and decreased with increasing concentration when the concentration of sodium pyruvate was higher than 25 g/L. The lycopene titer reached a maximum of 18.16 mg/L (4.13 mg/g DCW) at 36 h at a concentration of 25 g/L of sodium pyruvate, while the OD_600_ of strain H0 also reached a maximum of 12.57 (Fig. S3b). Nitrogen sources screening was performed by supplementing 2 g/L of candidate compounds (beef extract, corn steep liquor, urea, ammonium oxalate, ammonium sulfate, ammonium chloride) to the basal medium. As shown in Fig. S3c, the control group without any other nitrogen source exhibited the highest lycopene production at 18.42 mg/L (4.67 mg/g DCW). These results indicate that the complex mixture of yeast extract and tryptone in 2 YT medium provided an optimal nitrogen composition. Compared with the control, supplementation with any of the single nitrogen sources tested did not enhance lycopene production. Notably, inorganic nitrogen sources such as ammonium oxalate and ammonium chloride, strongly suppressed lycopene synthesis. In terms of cell growth, the OD_600_ with the addition of ammonium oxalate and ammonium chloride was not as high as that of the other groups. Lycopene titer and cellular biomass tended to decrease with increasing inoculum amount (1–5 % v/v). Using a 1 % inoculum amount, the highest lycopene titer (20.82 mg/L, 4.65 mg/g DCW) was achieved at 36 h, while peak biomass of 13.96 was reached at 48 h (Fig. S3d).

**Fig. 2:**
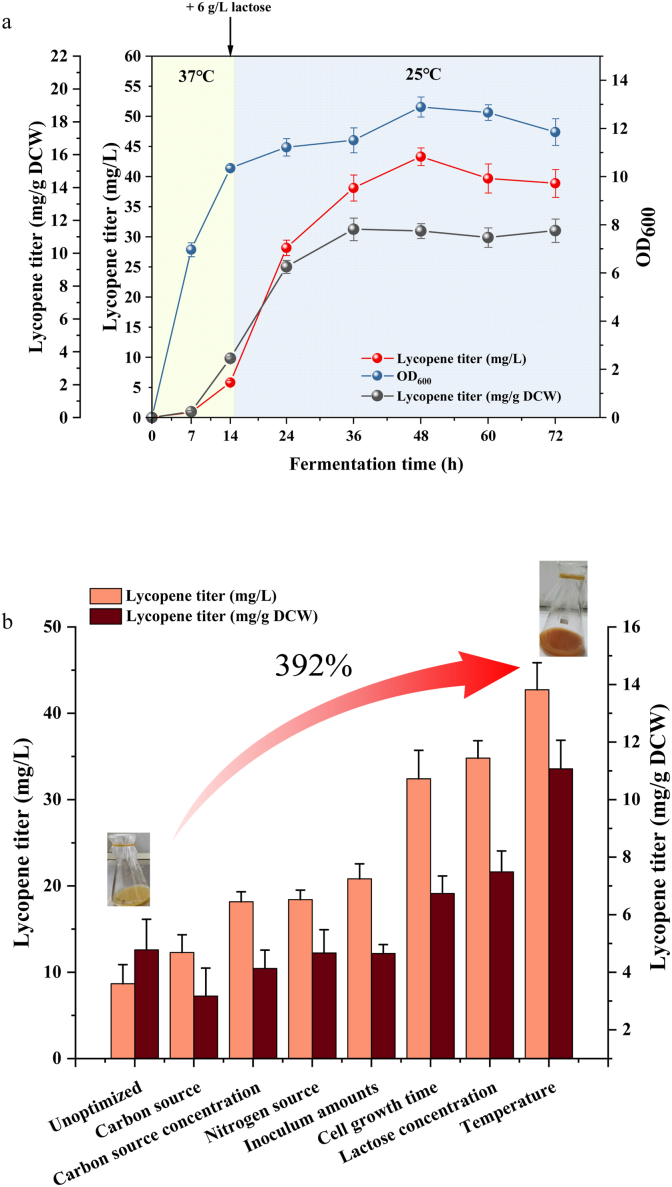
A two-stage fermentation optimization process strategy (a) Lycopene titer and biomass under optimal cultivation conditions; (b) Effect of optimization process on lycopene titer.

Sodium pyruvate, identified as a recently discovered effective carbon source for lycopene biosynthesis, significantly increased lycopene titer [22]. This is because pyruvate serves as dual roles, (1) the direct substrate for the upstream MEP pathway (Fig. 1), and (2) promoting cellular biomass, resulting in an increased lycopene titer. We speculate that it may be that the bacteria cannot grow sufficiently at too low a concentration of sodium pyruvate, while at too high a concentration they are affected by high substrate stress, which affects lycopene production. Notably, glucose exhibited a strong inhibitory effects on lycopene biosynthesis, potentially through the repression of T7 RNA polymerase in T7-inducible expression systems, thereby inhibiting the expression of the target protein [23]. Similarly, Lee et al. found that the addition of glucose to the medium strongly inhibited the production of carotenoids [24]. In addition, lactose also inhibits lycopene biosynthesis, this is because our fermentation process is induced by lactose, and glycolytic intermediates may interfere with lac operon activation, consequently suppressing heterologous gene expression [22]. Consistent with previous studies, including our own research, glycerol has been identified as the optimal carbon source for lycopene synthesis in recombinant *E*. *coli* [14,20,25,26], largely because it serves as the closest substance to G3P, the starting substrate of the upstream MEP pathway. In the present study, glycerol supplementation did not significantly enhance lycopene production. Furthermore, maltose was also observed to suppress lycopene production, which is consistent with the previous findings [22,[26], [27], [28]]. We speculate that the inhibitory effect of maltose may be attributed to carbon catabolite repression (CCR), a well-documented phenomenon in which readily metabolizable sugars such as glucose and other carbohydrates including glycerol, maltose, mannose, sucrose and xylose, negatively regulate the synthesis of secondary metabolites [29].

Inorganic nitrogen sources, specifically ammonium oxalate and ammonium chloride, inhibited both cell growth and lycopene production. Conversely, organic nitrogen sources enhanced biomass productivity due to their complex nutrient composition including amino acids, peptides, and essential carbohydrate derivatives. Inorganic nitrogen sources (ammonium oxalate and ammonium chloride) are monocomponent and contain only nutrients that do not exceed the minimum requirements for growth, and are less efficiently assimilated [30]. In addition, microbial metabolism of inorganic nitrogen sources triggered progressive accumulation of ammonium ions that negatively affected bacterial growth and pigment biosynthesis [31].

The inoculum amount affects the growth rate of microorganisms and consequently influences the biosynthesis of the target product. Inadequate inoculum density can prolong the lag phase of the strain due to insufficient initial biomass, while excessive inoculum amounts accelerate the exponential growth of the strain, which leads to dissolved oxygen depletion in the early cultivation stages. In our study the effect of inoculum amount (1–5 %) on lycopene titer and dissolved oxygen concentration at different inoculum amount was evaluated. Dissolved oxygen measurements revealed that at 1 % inoculation, the DO level dropped to 0 % within 14 h of fermentation. Furthermore, as the inoculation level increased, the rate of decline in DO concentration accelerated. When the inoculum amount raised to 5 %, the DO had already decreased to 0 % by 10 h (Fig. S3e). Higher inoculum concentrations (≥2 %) resulted in rapid oxygen depletion during the early stages of inoculation, leading to metabolic suppression and growth stagnation in subsequent fermentation stages. In contrast, the slower decline in dissolved oxygen concentration at 1 % inoculum sustained microbial growth, thereby maximizing lycopene titer.

### 3.2 Application of a two-stage fermentation strategy for lycopene production in chassis strain H0

The effect of cell growth time on lycopene production in recombinant strain H0 was determined using a two-stage fermentation strategy. The first stage involved biomass accumulation at 37 °C culture (2–16 h), followed by the induction of second stage at 25 °C for product biosynthesis (Fig. S4a). Both OD_600_ and lycopene titer of H0 increased with the extension of the first stage incubation time. When the incubation time of the first stage was 14 h, the lycopene titer reached the maximum value of 32.41 mg/L (6.74 mg/g DCW) after 48 h of inoculation. Prolongation of the cell growth time of the first stage was further beyond 14 h resulted in lower lycopene titers, accompanied by reduced biomass accumulation.

In the second phase, the product accumulation phase, the cells grow slowly and synthesize lycopene using expressed enzymes to convert the substrates. Here we attempted to use lactose as an inducer (0–8 g/L) for lycopene production (Fig. S4b). In our prior investigations, we observed that lycopene production was enhanced in the absence of isopropyl-β-d-thiogalactoside (IPTG) induction [14,20]. Accordingly, IPTG was not added in this study and lactose was utilized as an alternative inducer. Due to the presence of leaky expression, lycopene could still be produced in the absence of IPTG and lactose induction [32]. The addition of lactose significantly improved lycopene biosynthesis but reduced cellular biomass compared to no addition of lactose. Lycopene titer increased gradually with increasing lactose concentration, and the biosynthesis of lycopene was most favoured at a lactose concentration of 6 g/L, with a lycopene titer of 34.80 mg/L (7.49 mg/g DCW). At this time, biomass accumulation reached a minimum under these conditions (OD_600_ = 13.28), indicating that the lactose concentration applied optimally balanced heterologous protein expression and lycopene biosynthesis. The effect of different induction temperatures (15 °C, 20 °C, 25 °C, 30 °C and 37 °C) on lycopene synthesis was investigated. The highest lycopene titer (42.74 mg/L and 11.07 mg/g DCW at 48 h) was obtained at an induction temperature of 25 °C (Fig. S4c). The cellular biomass was the highest of OD_600_ = 15.1 at 48 h at 37 °C, which is consistent with the optimal growth temperature of *E. coli*. When the induction temperature was 30 °C, both lycopene production and cellular biomass were lower than those at 25 °C.

Heterologous biosynthetic pathways often interfere with the growth and metabolism of the host microorganism, resulting in a critical trade-off between cell growth and product accumulation during fermentation. To address this, a two-stage fermentation strategy effectively separates microbial growth from the induction phases: engineered strains are first cultivated at optimal growth temperature for biomass accumulation, followed by low-temperature induction to activate target protein expression and product synthesis [33]. Our two-stage fermentation process enabled engineered strain H0 to achieve a maximum lycopene titer of 43.33 mg/L (11.36 mg/g DCW) after 48 h, which was a 3.9-fold enhancement compared to pre-optimization conditions (Fig. 2a). As shown in Fig. 2b, cell growth time and induction temperature exerted the most significant influence on lycopene titer during fermentation. Induction temperature critically influences fermentation by modulating recombinant enzyme expression, enzyme activities, and product accumulation [34]. Utilizing a temperature-regulated two-stage fermentation strategy (30 °C/26 °C), the α-bisabolene titer of engineered *Saccharomyces cerevisiae* was increased byapproximately 56.5 % compared to a single-temperature fermentation at 30 °C [35]. Wang et al. designed a dual-mode temperature-controlled switch allowing cell growth mode at temperatures above 37 °C and d-pantothenic acid production stage at 30 °C, effectively decoupling cell growth from d-pantothenic acid production in the engineered *E. coli* [36]. Induction timing is also very important. Early induction imposes metabolic burden that might hinder cell growth, while late induction leads to suboptimal product yield [37]. These examples demonstrate that two-stage fermentation strategy effectively alleviated the impact of toxic intermediates and metabolites on growth. By reducing metabolic burden during two-stage fermentation, it enabled simultaneous biomass and product accumulation, with results superior to those of outperforming single-temperature fermentation.

In the second phase of two-stage fermentation strategy, induction of enzyme expression is very important. Lactose can replace the expensive and toxic IPTG as a cheap inducer to induce target protein expression [38] and the accumulation of lycopene [22,32,39,40]. Without inducion, strains prioritize growth over metabolite synthsis. Beyond induction, lactose-derived intermediates may enter the MEP pathway, thereby increasing lycopene production [22]. While in our previous study, induction without IPTG proved effective [14], lactose induction yielded superior results here.

### 3.3 qPCR analysis of the effect of sodium pyruvate on gene expression levels

**Fig. 3:**
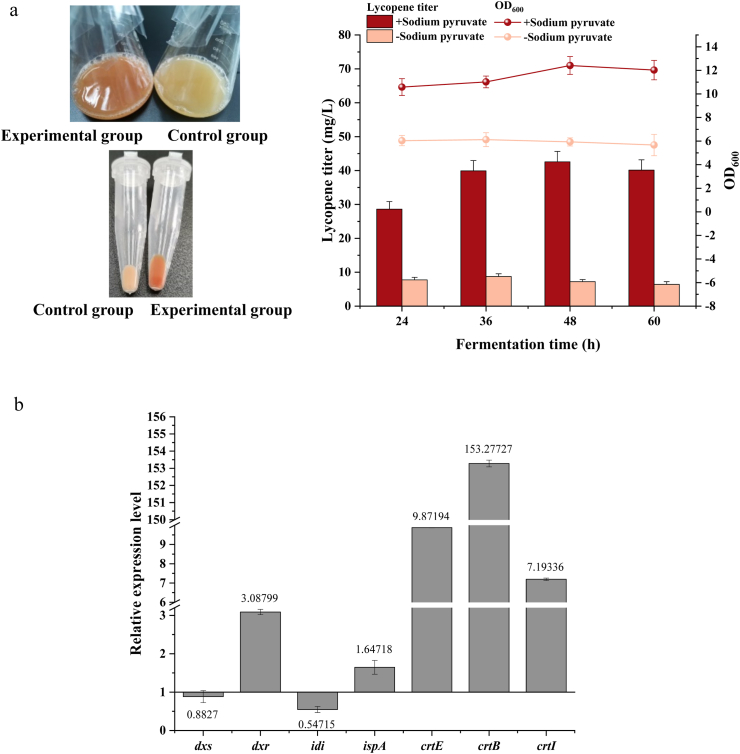
Effect of sodium pyruvate on lycopene yield and gene expression. (a) Lycopene production and cell density in experimental and control groups; (b) Relative expression level of *dxs*, *dxr*, *idi*, *ispA*, *crtE*, *crtB*, and *crtI* genes in the experimental group.

Supplementation with sodium pyruvate significantly increased lycopene titer and cell biomass (Fig. 3a). To isolate this effect, sodium pyruvate was evaluated as a single variable under otherwise constant conditions. Strain H0 was fermented with 25 g/L sodium pyruvate (the experimental group) versus no supplementation (the control group). Visual analysis of fermentation broth color and cellular precipitation (1 mL samples) clearly showed significant differences in lycopene production and biomass between the two groups (Fig. 3a). Both lycopene titer and biomass of the experimental group reached maximum at 48 h, substantially exceeding the control group. These results demonstrate that sodium pyruvate greatly promotes cell growth and metabolite accumulation, supporting its utility as a carbon source for lycopene production in engineered *E. coli*.

To investigate transcriptional expression differences in key genes under the two conditions, qPCR analysis was performed on 48-h fermentation cell pellets from the experimental and control groups to detect the expression levels of key genes in the MEP pathway and lycopene metabolism pathway (*dxs*, *dxr*, *idi*, *ispA*, *crtE*, *crtB* and *crtI*). The experimental group exhibited reduced relative transcriptional expression of *dxs* and *idi* genes (less than 1-fold), but elevated relative expression of *dxr*, *ispA*, *crtE*, *crtB* and *crtI* (more than 1-fold) compared to the control group (Fig. 3b). Notably, lycopene synthesis genes, *crtE*, *crtB* and *crtI*, showed significant upregulation, with the relative expression of *crtB* reaching 153.3-fold. Enhanced lycopene production could be due to the upregulation of *crtE/crtB/crtI*, which increases the levels of the corresponding enzymes.

qPCR analysis revealed a general upregulation of the downstream genes directly responsible for lycopene synthesis (*crtE*, *crtB*, *crtI*) as well as partial genes within the MEP pathway (*dxr*, *ispA*), which is consistent with the observed increase in lycopene production and indicates an effective channeling of carbon flux toward the target product. In contrast, the transcription of *dxs* and *idi*, which encode key rate-limiting enzymes in the pathway, was downregulated. This indicates gene expression changes that do not consistently correlate with changes and lycopene yield levels, suggesting additional regulatory mechanisms at post-transcriptional, enzymatic or metabolic flux levels. The reduction in transcription levels of *dxs* and *idi* may be attributed to the supplementation of sodium pyruvate, which partially alleviated the precursor supply pressure, thereby reducing the necessity for high levels of de novo synthesis. Furthermore, the downregulation may also result from the feedback inhibition and toxic effects arising from the accumulated IPP/DMAPP [10,41]. Furthermore, sodium pyruvate supplementation likely alters the intracellular phosphoenolpyruvate (PEP)/pyruvate ratio, influences cAMP production through the phosphotransferase system (PTS). The resulting cAMP-CRP complex then induces the expression of glucose-repressed genes otherwise repressed under carbon-sufficient conditions, a mechanism associated with carbon catabolite repression (CCR) [42,43]. It should also be noted that the sampling time point (48 h) corresponds to the late growth phase, during which *dxs* and *idi* transcription may have peaked earlier and subsequently declined as lycopene accumulation reached a plateau, thereby shifting the focus of regulation towards downstream genes (*crtE*, *crtB*, *crtI*). Given the low transcriptional levels of *idi* and *dxs* in H0 after 48 h, and considering that multiple strategies have been employed to successfully enhanced lycopene production by overexpressing these genes to increase metabolic flux through the MEP pathway [[44], [45], [46], [47]], we propose intensifying the overall metabolic process from upstream precursor supply within the MEP pathway to downstream product synthesis. This aims to further boost lycopene synthesis when sodium pyruvate is added.

### 3.4 Regulation of lycopene synthesis based on endogenous metabolic pathway

**Fig. 4:**
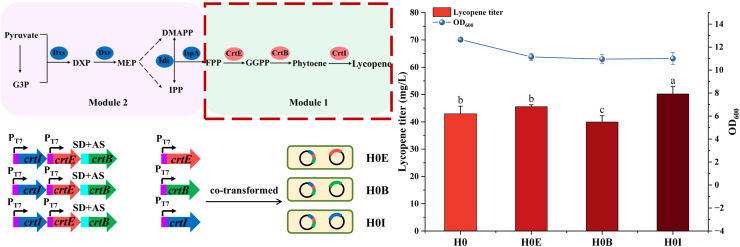
Effect of *crtE*, *crtB* and *crtI* overexpression on lycopene yield.

The overall lycopene metabolic pathway was divided into two modules of Module 1 (lycopene synthesis genes *crtE*, *crtB*, and *crtI*) and Module 2 (MEP pathway genes *dxs*, *dxr*, *idi*, and *ispA*) for further regulation (Fig. 4). qPCR analysis revealed sodium pyruvate supplementation upregulated *crtE*, *crtB* and *crtI* expression in Module 1. Consequently, we prioritized increasing the copy number of these terminal biosynthetic genes to enhance individual expression.

The *crtE*, *crtB* and *crtI* genes were cloned into plasmid pRSFDuet-1, generating pRSF-E, pRSF-B, pRSF-I, which were transformed into competent cells of H0 to obtain recombinant strains H0E, H0B and H0I, respectively. Strains H0E and H0I exhibited 1.06- and 1.17-fold higher elevated lycopene titers than H0, respectively, while H0B showed reduced production (Fig. 4). qPCR analysis revealed low basal expression of *crtE* and *crtI* genes in strain H0 (*crtE* > *crtI*), contrasting with high *crtB* expression. These results indicate insufficient *crtE* and *crtI* expression in chassis strain H0. Overexpressing these genes enhanced corresponding enzyme levels, thus increasing lycopene production. Conversely, the overexpression of *crtB* may cause a metabolic burden, thereby reducing the lycopene titers.

Gene copy number effects on lycopene synthesis (*crtE*, *crtB*, *crtI*) have been investigated. Schwartz et al. reported that additional overexpression of *crtI* in *Yarrowia lipolytica* harboring single-copy *crtEBI* significantly increased lycopene production, whereas overexpression of *crtE* and *crtB* showed no enhancement [48], which is consistent with our findings. This may result from intermediate accumulation. Similarly, Kang et al. demonstrated that *crtB* and *dxs* overexpression significantly increased lycopene titers in *crtLm*-knockout *Deinococcus radiodurans* R1, while *crtE* or *crtI* overexpression had no effect [49]. Jeong et al. overexpressed *dxs*, *idi*, *crtE* and *crtB* to produce phytoene in metabolically-engineered *D*. *radiodurans* R1 strain, respectively. Only *crtB* overexpression increased phytoene yield (*crtE*, *dxs* or *idi* gene showed no significant effects). Coexpression of *dxs* and *crtB* further enhanced phytoene production, whereas coexpression of *idi* + *crtB* and *crtE* + *crtB* decreased the phytoene yield [50]. These studies suggest that increasing the copy number of *crtE*, *crtB*, or *crtI* may increase lycopene titer. However, variations in metabolic profiles, enzymatic efficiency, and carbon fluxes in different microorganisms lead to differences in enzyme expression and lycopene titer.

### 3.5 Regulation of lycopene synthesis based on endogenous MEP pathway

**Fig. 5:**
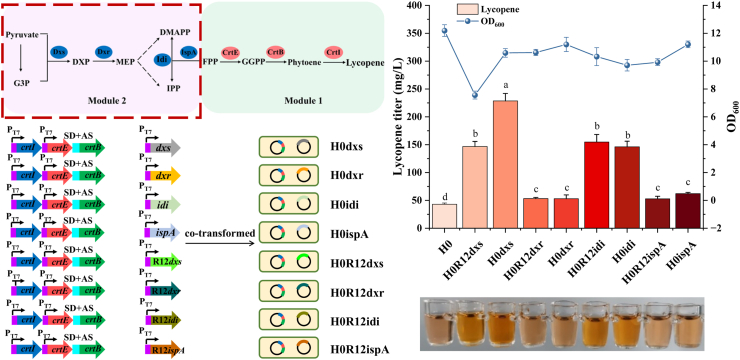
Fermentation data and acetone extracts of strains H0, H0R12dxs, H0dxs, H0R12dxr, H0dxr, H0R12idi, H0idi, H0R12ispA, H0ispA.

In the MEP pathway, *dxs*, *dxr*, *idi*, and *ispA* genes have been demonstrated to regulate lycopene synthesis [2,51]. In order to investigate their overexpression effects, the *dxs*, *dxr*, *idi* and *ispA* derived from *D. wulumuqiensis* R12 and *E. coli* MG1655 were individually cloned into pRSFDuet-1 to form pRSF-R12dxs, pRSF-R12dxr, pRSF-R12idi, pRSF-R12ispA, pRSF-dxs, pRSF-dxr, pRSF-idi, and pRSF-ispA, respectively. Then these recombinant plasmids were transformed into chassis strain H0 to obtain eight recombinant strains H0R12dxs, H0R12dxr, H0R12idi, H0R12ispA, H0dxs, H0dxr, H0idi, and H0ispA. Compared to the original strain H0 (42.93 mg/L, 11.06 mg/g DCW), all eight strains showed increased lycopene titers but decreased cell biomass at 48 h (Fig. 5). It is noteworthy that overexpression of *dxs* originating from *E. coli* in H0 resulted in the highest lycopene titer. Strains H0R12dxs and H0dxs achieved lycopene titers of 146.76 (55.47 mg/g DCW) and 228.46 mg/L (61.66 mg/g DCW), respectively, which were 3.42 and 5.32 times higher than that of chassis strain H0. In addition, similar to overexpression of *dxs*, elevated *idi* expression significantly enhanced lycopene production. Strains H0R12idi and H0idi achieved lycopene titers of 154.76 (42.85 mg/g DCW) and 146.20 mg/L (43.06 mg/g DCW), representing 3.60- and 3.41-fold increases over the chassis strain H0, respectively.

**Fig. 6:**
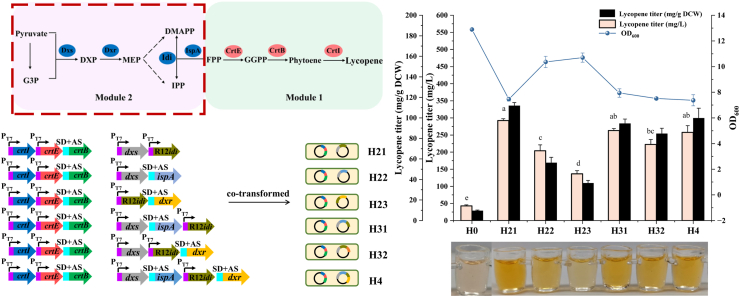
Fermentation data and acetone extracts of strains H0, H21, H22, H23, H31, H32, H4.

In comparison with the ability of each gene source to increase lycopene production, the optimal gene variants selected were *dxs*, *dxr* and *ispA* from *E. coli* MG1655 and *idi* gene from R12. These genes were combinatorially co-overexpressed (Table S3) to explore their synergistic effect on lycopene production (Fig. 6). All genetic configurations significantly increased lycopene titers relative to original strain H0. Furthermore, combinatorial overexpressions of *dxs-R12idi*, *dxs-ispA-R12idi*, and *dxs-ispA-R12idi-dxr* increased lycopene titers by 292.10, 262.88, and 257.53 mg/L (111.87, 94.52 and 99.79 mg/g DCW), respectively. Among them, the combined overexpression of *dxs-R12idi* gene combination was found to have the most significant effect, with the highest lycopene titer being obtained. The optimal lycopene-producing strain H21 was cultivated in shake flasks (Fig. S5). Lycopene titers reached a maximum of 293.70 mg/L (112.49 mg/g DCW) at 48 h. OD_600_ measurements indicated that growth inhibition began after 24 h, suggesting that accumulated lycopene may be toxic to the cells and inhibit growth. Therefore, future work should focus on mitigating product toxicity to further improve the lycopene titer. As shown in Fig. S6, after ten rounds of subcultivation, the lycopene titer produced by strain H21 ranged between 293 and 266 mg/L. Plasmid stability gradually decreased from 99.51 % to 80.86 %, but remained consistently above 80 %. These results indicate that recombinant strain H21 possesses both a robust capacity for lycopene biosynthesis and satisfactory plasmid stability, making it a suitable high-quality chassis for subsequent production or engineering modification.

Numerous studies have been reported in the literature on these four key genes in the MEP pathway. *dxs* and *idi* encode key rate-limiting enzymes in the MEP pathway [2]. DXS (encoded by *dxs*), a thiamine-dependent enzyme, catalyzes the first step of the MEP pathway (Fig. 1). In vitro studies indicate that DXS activity in some bacteria is inhibited by negative feedback regulation of MEP pathway products (IPP and DMAPP) [41]. The overexpression of *dxs* critically regulates metabolic fluxes and intermediates in the MEP and lycopene pathway [5,24,44]. Furthermore, heterologous *dxs* expression from diverse sources (e.g., *D. radiodurans* R1, *Rhodobacter sphaeroides*, *Zymomonas mobilis*, *B. subtilis*, *Salvia pomifera*, *Rhodopseudomonas palustris*) expanded and broadened the types of genetically engineered hosts for lycopene or isoprenoid production [45,[52], [53], [54], [55]].

The primary role of IDI (encoded by *idi*) likely involves balancing cellular IPP and DMAPP pools, as excessive levels of either isoprenoid precursor can be harmful to bacterial growth. However, in *E. coli* native chromosomal *idi* gene expression is repressed [56], which has a detrimental impact on lycopene biosynthesis. Consequently, cloning *idi* into an expression vector can enhance its expression and thereby increase lycopene production. In addition to traditional gene expression strategies, recent efforts have focused on diversifying *idi* gene sources and genomic integration using tools such as CRISPR-Cas9. For instance, exogenous *idi* genes from *D*. *radiodurans*, *Deinococcus radiopugnans*, and *Myxococcus xanthus* were screened and immobilized on the carboxysome nanocages resulted in the higher yield of lycopene compared to strains with unimmobilized IDI in *E. coli* [57]. Heterologous expression of *idi* genes from *E. coli* and *B. subtilis* all increased lycopene cell-specific productivity in *E. coli* by 95 %, 93 %, and 109 %, respectively, compared to the control strain [46]. Similarly, overexpression of *idi* gene from *C*. *glutamicum* in *Rhodococcus jostii* PET increased lycopene production by 44 % [47]. The expression of codon-optimized *idi* from *S*. *cerevisiae* in strain RPLYC5 yielded 6.90 mg/g DCW lycopene, a 2.12-fold increase compared to the wild-type strain [45]. CRISPR/Cas9-mediated integration of the artificial cluster *ispG*-*idi* into the *B. subtilis* CrtEIB-IspA chromosome and expression elevated lycopene titer by 39.3 % compared to the original strain [58].

While the expression of *dxr* and *ispA* genes was less effective than that of *dxs* and *idi* in enhancing lycopene production [56,59], it nevertheless exerted a positive influence on lycopene and terpene biosynthesis. For instance, Yan et al. demonstrated that introducing *dxr* from *Salvia miltiorrhiza* into *E. coli* increased lycopene production [60]. Similarly, overexpressing the endogenous *dxr* gene from *C*. *glutamicum* significantly enhanced terpene synthesis, elevating α-farnesene production 9-fold relative to the wild-type control [51]. Typically, co-expressing these two genes with other rate-limiting enzymes in the MEP pathway yields superior results.

In multienzyme pathways, overexpressing individual or more enzymes can lead to metabolic imbalances between upstream and downstream modules. This can cause toxic intermediate accumulation, inhibiting cell growth and product synthesis, thereby creating new metabolic bottlenecks. In *E. coli*, combinatorial expression of the key enzymes in MEP pathway has successfully enhanced terpenoid production. Production of isoprene, valencene, α-farnesene, lycopene, β-carotene and astaxanthin has also been improved using the combinatorial expression of key enzymes including *dxs*-*idi* [6,7,44,47,51], *dxs-ispA* [24], *dxs*-*dxr* [61], *idi*-*ispA* [62], *ispA*-*idi*-*dxs* [59], *dxs*-*dxr*-*idi* [63], *idsA*-*idi*-*crtB*-*crtI* [64], and *dxs*-*dxr*-*idi*-*ispA* [18]. Various studies consistently demonstrate that co-expression of *dxs* and *idi* genes significantly enhances carotenoid production, though effects vary with gene sources. Among tested combinations in this study, the highest lycopene titer was generated by overexpressing *dxs* from *E*. *coli* MG1655 and *idi* from R12, which exceeded the results of single-gene overexpression. Consequently, strain H21 was selected for optimal lycopene production. We hypothesize this improvement stems from: (i) enhanced precursors supply in MEP pathway via *dxs* overexpression; (ii) balanced IPP and DMAPP pools via *idi* overexpression; and (iii) alleviatied feedback inhibition of DXS catalytic activity by IPP and DMAPP through combined *dxs*-*idi* expression [65].

Table 1 summarizes regulatory strategies employed for lycopene production in *E*. *coli*, including diverse gene sources, enhancement of MEP pathway, introduction of MVA pathway, and recently emerging new technologies. These strategies have effectively improved lycopene yields. In our study, the combined strategy of sodium pyruvate supplementation and regulation of the endogenous MEP pathway enabled lycopene titer to reach 293.70 mg/L (112.49 mg/g DCW). First, this titer represents a 33.88-fold increase compared with the unoptimized initial strain H0 (8.67 mg/L) and exceeds most of the titers reported in Table 1, demonstrating the effectiveness of both sodium pyruvate addition and MEP pathway modulation. Second, while introducing heterologous mevalonate (MVA) pathways from *Saccharomyces cerevisiae* or other sources (e.g., Introduction of MVA pathway in Table 1) can achieve high titers—typically in the range of 100–350 mg/L—such approaches generally require the introduction of 5–8 heterologous genes, imposing considerable genetic and metabolic burden on the host. In contrast, our method involves only the precise upregulation of three key MEP pathway genes (*dxs*, *idi*, *ispA*) together with the core *crt* genes, leveraging the synergy with the prokaryotic endogenous MEP pathway to enhance lycopene synthesis. Finally, our titer is also superior to those obtained with emerging strategies such as cell-free systems or phase separation, while optimized endogenous pathways based on fermentation systems demonstrate distinct advantages in terms of cost-effectiveness and engineering potential. Therefore, by combining endogenous MEP pathway regulation with the promotive effect of sodium pyruvate, this study achieved a high level of lycopene production. Additionally, the high-yield strain H21 developed in this study can serve as an excellent chassis for the synthesis of other similar carotenoids. This approach provides a reference for establishing a multienzyme synthesis system for lycopene and its genetic-level regulation. Future research offers significant scope for refinement. Through promoter engineering, host screening, membrane engineering, lipid metabolism modulation, and compartmentalization, lycopene yields in *E*. *coli* can be further enhanced.

**Table 1:** Overview of metabolic engineering optimization strategies for lycopene production in *E*. *coli*.

Main methods	Specific regulatory strategies	Yield/Titer	Ref.
Diverse gene sources	immobilized exogenous *idi* genes from *D*. *radiodurans*, *Deinococcus radiopugnans*, and *Myxococcus xanthus* on the carboxysome nanocages	10 mg/g DCW	[57]
	overexpression of *dxs* and *ispA* from *Vibrio* sp. Dhg	20.3 mg/g DCW	[16]
	using *crtE, crtB,* and *crtI* from *Pantoea agglomerans* and *Pantoea ananatis*, *gps* from*Archaeoglobus fulgidus*	60 mg/L	[15]
Enhancement of MEP pathway	knockout of *zwf* and overexpression of *idi*, *dxs*, *ispD*, *ispF*	7.55 mg/g DCW	[6]
	modulated *sdhABCD*, *talB*, *dxs*, *idi*	20.05 mg/g DCW	[7]
	directed co-evolution of enzymes encoded by *dxs*, *dxr*, *idi*	0.65 mg/L	[8]
Introduction of MVA pathway	the copy number and integrated position of two modules of mevalonate (MVA) pathway and one module of lycopene expression pathway were optimized and integrated in the chromosome	393.24 mg/L	[9]
	The MVA pathway was introduced and adjusted using the ribosome binding sites (RBSs) library	219.7 mg/g DCW	[10]
	The MVA pathway was modified with araBAD promoter and constitutive 119 promoter using glucose as substrate	0.15 g/L	[23]
Enhancement of MEP pathway and diverse gene sources	integrated the exogenous *crtEBI* genes from *C. glutamicum*, deleted *aceE* and *gdhA* and overexpressed four genes involving in MEP pathway (*dxs*, *dxr*, *ispA* and *idi*)	25.82 mg/g DCW	[17]
	the heterogenous *crtEBI* from *C*. *glutamicum* and *dxs*, *dxr*, and *ispA* from *E. coli* were overexpressed in 10 LPS mutant *E. coli*	5.39 mg/g DCW	[18]
Introduction of MVA pathway and diverse gene sources	introduced the MVA pathway with genes from *Enterococcus saccharolyticus*, *S. cerevisiae* and *B. subtilis*	77 mg/L	[46]
	the heterologous mevalonate pathway and *crtEBI* genes from *C*. *glutamicum* were overexpressed and membrane permeability was improved	78.92 mg/L	[11]
New technology	optimized a CRISPR-Cas genome engineering protocol for knocking out 16 genes and combining 7 deletions	135 mg/L	[12]
	immobilized ReverseTag_Dxr, ReverseTag_Dxs, and ReverseTag_Idi by forming ester bonds with ReverseCatcher_RGGRGG	108 mg/L	[39]
	lycopene was synthesized in vitro by mixing the crude extract containing the expressed three enzymes GGPPS, PSY and PDS in a cell-free system	14.06 mg/L	[66]
	AtoB, HmgS, and HmgR for lycopene biosynthesis have been co-localized on the exterior of the engineered protein cages	151.6 mg/L	[67]
Enhancement of MEP pathway and diverse gene sources	optimized combination of *crtE*, *crtB*, *crtI*, *idi* from *D*. *wulumuqiensis* R12 and *dxs* from *E. coli* MG1655 with the addition of sodium pyruvate	293.70 mg/L 112.49 mg/g DCW	This study

In this study, the lycopene pathway from *D. wulumuqiensis* R12 strain was reconstructed in *E*. *coli*, generating the chassis strain H0 for heterologous synthesis of lycopene. Subsequent medium and fermentation optimization revealed sodium pyruvate enhanced lycopene accumulation. qPCR analysis demonstrated that sodium pyruvate increased the expression levels of *dxr*, *ispA*, *crtE*, *crtB* and *crtI*, while decreasing the expression of *dxs* and *idi*, prompting endogenous pathway engineering. The lycopene biosynthetic pathway and native MEP pathway in *E. coli* were engineered through employing the overexpression strategy of increasing gene copy numbers. The *dxs*, *dxr* and *ispA* from *E. coli* MG1655 and *idi* from *D. wulumuqiensis* R12 were screened and identified as advantageous genes. Combinatorial co-expression of advantageous genes demonstrated that *dxs* gene of *E. coli* MG1655 with *idi* gene of *D. wulumuqiensis* R12 was the most effective combination for synthesis of lycopene. Strain H21 produced 293.70 mg/L of lycopene, representing a 33.88-fold increase compared to the chassis strain H0.

## CRediT authorship contribution statement

**Xian Xu:** Writing – review & editing, Project administration, Funding acquisition, Conceptualization. **Hongyu Xing:** Writing – original draft, Methodology, Investigation. **Hui Zhi:** Methodology, Investigation, Data curation. **Chen Qin:** Validation, Investigation, Data curation. **Yuyue Deng:** Methodology, Investigation. **Wanqi Wei:** Methodology, Investigation. **Chunyan Huang:** Writing – review & editing, Methodology, Investigation, Conceptualization.

## Declaration of competing interest

The authors declare that they have no known competing financial interests or personal relationships that could have appeared to influence the work reported in this paper.

## Data Availability

Data will be made available on request.

## References

[1] Wu H., Wu Y., Cui Z., Hu L. (2024). Nutraceutical delivery systems to improve the bioaccessibility and bioavailability of lycopene: a review. Crit Rev Food Sci Nutr.

[2] Du B., Sun M., Hui W., Xie C., Xu X. (2023). Recent advances on key enzymes of microbial origin in the lycopene biosynthesis pathway. J Agric Food Chem.

[3] Li L., Liu Z., Jiang H., Mao X. (2020). Biotechnological production of lycopene by microorganisms. Appl Microbiol Biotechnol.

[4] Rinaldi M.A., Ferraz C.A., Scrutton N.S. (2022). Alternative metabolic pathways and strategies to high-titre terpenoid production in *Escherichia coli*. Nat Prod Rep.

[5] Kim S.W., Keasling J.D. (2001). Metabolic engineering of the nonmevalonate isopentenyl diphosphate synthesis pathway in *Escherichia coli* enhances lycopene production. Biotechnol Bioeng.

[6] Zhou Y., Nambou K., Wei L., Cao J., Imanaka T., Hua Q. (2013). Lycopene production in recombinant strains of *Escherichia coli* is improved by knockout of the central carbon metabolism gene coding for glucose-6-phosphate dehydrogenase. Biotechnol Lett.

[7] Jiang R., Chen X., Lian J., Huang L., Cai J., Xu Z. (2019). Efficient production of pseudoionone with multipathway engineering in *Escherichia coli*. J Appl Microbiol.

[8] Lv X., Gu J., Wang F., XieW Liu M., Ye L., Yu H. (2016). Combinatorial pathway optimization in *Escherichia coli* by directed co‐evolution of rate‐limiting enzymes and modular pathway engineering. Biotechnol Bioeng.

[9] Hussain M.H., Hong Q., Zaman W.Q., Mohsin A., Wei Y., Zhang N., Fang H., Wang Z., Hang H., Zhuang Y. (2021). Rationally optimized generation of integrated *Escherichia coli* with stable and high yield lycopene biosynthesis from heterologous mevalonate (MVA) and lycopene expression pathways. Synth Syst Biotechnol.

[10] Cheng T., Wang L., Sun C., Xie C. (2022). Optimizing the downstream MVA pathway using a combination optimization strategy to increase lycopene yield in *Escherichia coli*. Microb Cell Fact.

[11] Fordjour E., Bai Z., Li S., Li S., Sackey I., Yang Y., Liu C.L. (2023). Improved membrane permeability via hypervesiculation for in situ recovery of lycopene in *Escherichia coli*. ACS Synth Biol.

[12] Shukal S., Lim X.H., Zhang C., Chen X. (2022). Metabolic engineering of *Escherichia coli* BL21 strain using simplified CRISPR-Cas9 and asymmetric homology arms recombineering. Microb Cell Fact.

[13] Wang Y., San K.Y., Bennett G.N. (2013). Improvement of NADPH bioavailability in *Escherichia coli* through the use of phosphofructokinase deficient strains. Appl Microbiol Biotechnol.

[14] Xu X., Tian L., Xu J., Xie C., Jiang L., Huang H. (2018). Analysis and expression of the carotenoid biosynthesis genes from *Deinococcus wulumuqiensis* R12 in engineered *Escherichia coli*. AMB Express.

[15] Yoon S.H., Kim J.E., Lee S.H., Park H.M., Choi M.S., Kim J.Y., Lee S.H., Shin Y.C., Keasling J.D., Kim S.W. (2007). Engineering the lycopene synthetic pathway in *E. coli* by comparison of the carotenoid genes of *Pantoea agglomerans* and *Pantoea ananatis*. Appl Microbiol Biotechnol.

[16] Kim M.J., Noh M.H., Woo S., Lim H.G., Jung G.Y. (2019). Enhanced lycopene production in *Escherichia coli* by expression of two MEP pathway enzymes from *Vibrio* sp. dhg. Catalysts.

[17] Hu X., Cui M., Wang X. (2023). Improvement of lycopene biosynthesis in waaC and waaF mutants of *Escherichia coli* by integrant expression of crtEBI gene and deletion of aceE and gdhA. Syst Microbiol Biomanuf.

[18] Cui M., Wang Z., Hu X., Wang X. (2019). Effects of lipopolysaccharide structure on lycopene production in *Escherichia coli*. Enzym Microb Technol.

[19] Liu Y.S., Wu J.Y., Ho Kp (2006). Characterization of oxygen transfer conditions and their effects on *Phaffia rhodozyma* growth and carotenoid production in shake-flask cultures. Biochem Eng J.

[20] Xu X., Jin W., Jiang L., Xu Q., Li S., Zhang Z., Huang H. (2016). A high-throughput screening method for identifying lycopene-overproducing *E. coli* strain based on an antioxidant capacity assay. Biochem Eng J.

[21] Zhou K., Zhou L., Lim Q., Zou R., Stephanopoulos G., Too H.P. (2011). Novel reference genes for quantifying transcriptional responses of *Escherichia coli* to protein overexpression by quantitative PCR. BMC Mol Biol.

[22] Chen Y., Ming D., Zhu L., Huang H., Jiang L. (2022). Tailoring the tag/catcher system by integrating covalent bonds and noncovalent interactions for highly efficient protein self-assembly. Biomacromolecules.

[23] Liu N., Liu B., Wang G., Soong Y.-H.V., Tao Y., Liu W., Xie D. (2020). Lycopene production from glucose, fatty acid and waste cooking oil by metabolically engineered *Escherichia coli*. Biochem Eng J.

[24] Lee P.C., Mijts B.N., Schmidt-Dannert C. (2004). Investigation of factors influencing production of the monocyclic carotenoid torulene in metabolically engineered *Escherichia coli*. Appl Microbiol Biotechnol.

[25] Kim J., Kong M.K., Lee S.Y., Lee P.C. (2010). Carbon sources-dependent carotenoid production in metabolically engineered *Escherichia coli*. World J Microbiol Biotechnol.

[26] Yoon S.H., Lee S.H., Das A., Ryu H.K., Jang H.J., Kim J.Y., Oh D.K., Keasling J.D., Kim S.W. (2009). Combinatorial expression of bacterial whole mevalonate pathway for the production of β-carotene in *E. coli*. J Biotechnol.

[27] Farmer W.R., Liao J.C. (2001). Precursor balancing for metabolic engineering of lycopene production in *Escherichia coli*. Biotechnol Prog.

[28] Marcoleta A., Niklitschek M., Wozniak A., Lozano C., Alcaíno J., Baeza M., Cifuentes V. (2011). Glucose and ethanol-dependent transcriptional regulation of the astaxanthin biosynthesis pathway in *Xanthophyllomyces dendrorhous*. BMC Microbiol.

[29] Ruiz B., Chávez A., Forero A., García-Huante Y., Romero A., Sánchez M., Rocha D., Sánchez B., Rodríguez-Sanoja R., Sánchez S. (2010). Production of microbial secondary metabolites: regulation by the carbon source. Crit Rev Microbiol.

[30] Ma Y., Sun Z., Yang H., Xie W., Song M., Zhang B., Sui L. (2024). The biosynthesis mechanism of bacterioruberin in halophilic archaea revealed by genome and transcriptome analysis. Appl Environ Microbiol.

[31] Minyuk G., Sidorov R., Solovchenko A. (2020). Effect of nitrogen source on the growth, lipid, and valuable carotenoid production in the green microalga *Chromochloris zofingiensis*. J Appl Phycol.

[32] Li Y., Guo Q., Zhang T., Wang C., Yang H., Du G., Li R. (2022). Measurement of lactose concentration in milk by using engineered bacteria producing lycopene. J Microbiol Methods.

[33] Shabestary K., Klamt S., Link H., Mahadevan R., Steuer R., Hudson E.P. (2024). Design of microbial catalysts for two-stage processes. Nat Rev Bioeng.

[34] Daniel R.M., Danson M.J. (2013). Temperature and the catalytic activity of enzymes: a fresh understanding. FEBS Lett.

[35] Feng P., Sun B., Bi H., Bao Y., Wang M., Zhang H., Fang Y. (2025). Developing thermosensitive metabolic regulation strategies in the fermentation process of *Saccharomyces cerevisiae* to enhance α-bisabolene production. ACS Synth Biol.

[36] Wang Y., Zhou J., Zhang Z., Huang L., Zhang B., Liu Z., Zheng Y. (2024). Efficient carbon flux allocation towards D-pantothenic acid production via growth-decoupled strategy in *Escherichia coli*. Bioresour Technol.

[37] De Baets J., De Paepe B., De Mey M. (2024). Delaying production with prokaryotic inducible expression systems. Microb Cell Fact.

[38] Matera A., Dulak K., Sordon S., Huszcza E., Popłoński J. (2025). Modular plasmid design for autonomous multi-protein expression in *Escherichia coli*. J Biol Eng.

[39] Chen Y., Shi Y., Li M., Ming D., Liu W., Xu X., Jiang L. (2025). Phase separation-mediated multienzyme assembly in vivo. J Agric Food Chem.

[40] Lautier T., Smith D.J., Yang L.K., Chen X., Zhang C., Truan G., Lindley N.D. (2023). β-Cryptoxanthin production in *Escherichia coli* by optimization of the cytochrome P450 CYP97H1 activity. J Agric Food Chem.

[41] Di X., Ortega-Alarcon D., Kakumanu R., Iglesias-Fernandez J., Diaz L., Baidoo E.E., Velazquez-Campoy A., Rodríguez-Concepción M., Perez-Gil J. (2023). MEP pathway products allosterically promote monomerization of deoxy-D-xylulose-5-phosphate synthase to feedback-regulate their supply. Plant Commun.

[42] Kremling A., Geiselmann J., Ropers D., de Jong H. (2015). Understanding carbon catabolite repression in *Escherichia coli* using quantitative models. Trends Microbiol.

[43] Schubert C., Unden G. (2024). Regulation of aerobic succinate transporter dctA of *E. coli* by cAMP-CRP, DcuS-DcuR, and EIIAGlc: succinate as a carbon substrate and signaling molecule. Microb Physiol.

[44] Khana D.B., Tatli M., Rivera Vazquez J., Weraduwage S.M., Stern N., Hebert A.S., Angelica Trujillo E., Stevenson D.M., Coon J.J., Sharky T.D. (2023). Systematic analysis of metabolic bottlenecks in the methylerythritol 4-phosphate (MEP) pathway of *Zymomonas mobilis*. mSystems.

[45] Li M., Xia Q., Lv S., Tong J., Wang Z., Nie Q., Yang J. (2022). Enhanced CO_2_ capture for photosynthetic lycopene production in engineered *Rhodopseudomonas palustris*, a purple nonsulfur bacterium. Green Chem.

[46] Kang M.K., Nguyen M.P., Yoon S.H., Jayasundera K.B., Son J.W., Wang C., Kwon M., Kim S.W. (2024). Reconstitution of the mevalonate pathway for improvement of isoprenoid production and industrial applicability in *Escherichia coli*. J Microb Biotechnol.

[47] Diao J., Hu Y., Tian Y., Carr R., Moon T.S. (2023). Upcycling of poly (ethylene terephthalate) to produce high-value bio-products. Cell Rep.

[48] Schwartz C., Frogue K., Misa J., Wheeldon I. (2017). Host and pathway engineering for enhanced lycopene biosynthesis in *Yarrowia lipolytica*. Front Microbiol.

[49] Kang C.K., Jeong S.W., Yang J.E., Choi Y.J. (2020). High-yield production of lycopene from corn steep liquor and glycerol using the metabolically engineered *Deinococcus radiodurans* R1 Strain. J Agric Food Chem.

[50] Jeong S.W., Kang C.K., Choi Y.J. (2018). Metabolic engineering of *Deinococcus radiodurans* for the production of phytoene. J Microbiol Biotechnol.

[51] Lim H., Park J., Woo H.M. (2020). Overexpression of the key enzymes in the methylerythritol 4-phosphate pathway in *Corynebacterium glutamicum* for improving farnesyl diphosphate-derived terpene production. J Agric Food Chem.

[52] Wu X., Ma G., Liu C., Qiu Xy, Min L., Kuang J., Zhu L. (2021). Biosynthesis of pinene in purple non-sulfur photosynthetic bacteria. Microb Cell Fact.

[53] Zhao Y., Yang J., Qin B., Li Y., Sun Y., Su S., Xian M. (2011). Biosynthesis of isoprene in *Escherichia coli* via methylerythritol phosphate (MEP) pathway. Appl Microbiol Biotechnol.

[54] Einhaus A., Steube J., Freudenberg R.A., Barczyk J., Baier T., Kruse O. (2022). Engineering a powerful green cell factory for robust photoautotrophic diterpenoid production. Metab Eng.

[55] Su A., Chi S., Li Y., Tan S., Qiang S., Chen Z., Meng Y. (2018). Metabolic redesign of *Rhodobacter sphaeroides* for lycopene production. J Agric Food Chem.

[56] Yoon S.H., Lee Y.M., Kim J.E., Lee S.H., Lee J.H., Kim J.Y., Jung K.H., Shin Y.C., Keasling J.D., Kim S.W. (2006). Enhanced lycopene production in *Escherichia coli* engineered to synthesize isopentenyl diphosphate and dimethylallyl diphosphate from mevalonate. Biotechnol Bioeng.

[57] Zhou Y., Yao Y., Zhang F., Yu N., Wang B., Tian B. (2025). Enhancement of lycopene biosynthesis using self-assembled multi-enzymic protein cages. Microorganisms.

[58] Liu Y., Cheng H., Li H., Zhang Y., Wang M. (2023). A programmable CRISPR/Cas9 toolkit improves lycopene production in *Bacillus subtilis*. Appl Environ Microbiol.

[59] Sun J., Xu X., Wu Y., Sun H., Luan G., Lu X. (2023). Conversion of carbon dioxide into valencene and other sesquiterpenes with metabolic engineered *Synechocystis* sp. PCC 6803 cell factories. GCB Bioenergy.

[60] Yan X., Zhang L., Wang J., Liao P., Zhang Y., Zhang R., Kai G. (2009). Molecular characterization and expression of 1-deoxy-d-xylulose 5-phosphate reductoisomerase (DXR) gene from *Salvia miltiorrhiza*. Acta Physiol Plant.

[61] Hernandez-Arranz S., Perez-Gil J., Marshall-Sabey D., Rodriguez-Concepcion M. (2019). Engineering *Pseudomonas putida* for isoprenoid production by manipulating endogenous and shunt pathways supplying precursors. Microb Cell Fact.

[62] Göttl V.L., Meyer F., Schmitt I., Persicke M., Peters-Wendisch P., Wendisch V.F., Henke N.A. (2024). Enhancing astaxanthin biosynthesis and pathway expansion towards glycosylated C40 carotenoids by *Corynebacterium glutamicum*. Sci Rep.

[63] Lv X., Xu H., Yu H. (2013). Significantly enhanced production of isoprene by ordered coexpression of genes dxs, dxr, and idi in *Escherichia coli*. Appl Microbiol Biotechnol.

[64] Zhan Z., Chen X., Ye Z., Zhao M., Li C., Gao S., Sinskey A.J., Yao L., Dai J., Jiang Y. (2024). Expanding the CRISPR toolbox for engineering lycopene biosynthesis in *Corynebacterium glutamicum*. Microorganisms.

[65] Diao J., Tian Y., Hu Y., Moon T.S. (2025). Producing multiple chemicals through biological upcycling of waste poly (ethylene terephthalate). Trends Biotechnol.

[66] Chen S., Zhu G., Wang Z., Fu B., Sun M., Liu J., Xie C., Xu X. (2025). Construction and optimization of a cell-free metabolic engineering system for lycopene synthesis. Biochem Eng J.

[67] Kang W., Ma X., Kakarla D., Zhang H., Fang Y., Chen B., Zhu K., Zheng D., Wu Z., Li B. (2022). Organizing enzymes on self-assembled protein cages for cascade reactions. Angew Chem Int Ed.

