# Supplementary material for: Metabolic engineering of endogenous MEP pathway for enhanced lycopene production in Escherichia coli
Source: Synth Syst Biotechnol. 2026 Jan 30;13:109–19. doi: 10.1016/j.synbio.2026.01.014 (PMC12874350; doi:10.1016/j.synbio.2026.01.014)
Supplement: Multimedia component 1 [file mmc1.docx]

**Supplementary Information**

**Metabolic engineering of endogenous** **MEP pathway for enhanced lycopene production in *Escherichia coli***

Table S1 Plasmids and strains used in this work

| Plasmids/Strains | Features | Source |
| --- | --- | --- |
| pETDuet-1 | Amp^R^，T7 promoter | Invitrogen |
| pET-IEB | Amp^R^，pET-Duet-1 with the *crtE*、*crtB*、*crtI* genes of *D. wulumuqiensis* R12 | this study |
| pRSFDuet-1 | Kana^R^，T7 promoter | Novagen |
| pET22b-E | Amp^R^，pET-22b(+) with *crtE* gene from *D. wulumuqiensis* R12 | this study |
| pET22b-B | Amp^R^，pET-22b(+) with *crtB* gene from *D. wulumuqiensis* R12 | this study |
| pET22b-I | Amp^R^，pET-22b(+) with with *crtI* gene from *D. wulumuqiensis* R12 | this study |
| pET22b-R12idi | Amp^R^，pET-22b(+) with *idi* gene from *D. wulumuqiensis* R12 | this study |
| pET22b- R12ispA | Amp^R^，pET-22b(+)with *ispA* gene from *D. wulumuqiensis* R12 | this study |
| pRSF-E | Kana^R^，pRSFDuet-1 with *crtE* gene from *D. wulumuqiensis* R12 | this study |
| pRSF-B | Kana^R^，pRSFDuet-1 with *crtB* gene from *D. wulumuqiensis* R12 | this study |
| pRSF-I | Kana^R^，pRSFDuet-1 with *crtI* gene from *D. wulumuqiensis* R12 | this study |
| pRSF-dxs | Kana^R^，pRSFDuet-1 with *dxs* gene from *E.coli* MG1655 | this study |
| pRSF-dxr | Kana^R^，pRSFDuet-1 with *dxr* gene from *E.coli* MG1655 | this study |
| pRSF-idi | Kana^R^，pRSFDuet-1 with *idi* gene from *E.coli* MG1655 | this study |
| pRSF-ispA | Kana^R^，pRSFDuet-1 with *ispA* gene from *E. coli* MG1655 | this study |
| pRSF-R12dxs | Kana^R^，pRSFDuet-1 with *dxs* gene from *D. wulumuqiensis* R12 | this study |
| pRSF-R12dxr | Kana^R^，pRSFDuet-1 with *dxr* gene from *D. wulumuqiensis* R12 | this study |
| pRSF-R12idi | Kana^R^， pRSFDuet-1 with *idi* gene from *D. wulumuqiensis* R12 | this study |
| pRSF-R12ispA | Kana^R^，pRSFDuet-1 with *ispA* gene from *D. wulumuqiensis* R12 | this study |
| pRSF-dxsR12idi | Kana^R^，pRSF-Duet-1 with the *dxs* gene of *E.coli* MG1655 and the *idi* gene of *D. wulumuqiensis* R12 | this study |
| pRSF-dxsispA | Kana^R^，pRSF-Duet-1 with the *dxs* and *ispA* genes of *E.coli* MG1655 | this study |
| pRSF-R12ididxr | Kana^R^，pRSF-Duet-1 with the *dxr* gene of *E.coli* MG1655 and the *idi* gene of *D. wulumuqiensis* R12 | this study |
| pRSF-dxsispAR12idi | Kana^R^，pRSF-Duet-1 with the *dxs、ispA* genes of *E.coli* MG1655 and the *idi* gene of *D. wulumuqiensis* R12 | this study |
| pRSF-dxsispAR12idi | Kana^R^，pRSF-Duet-1 with the *dxs、dxr* genes of *E.coli* MG1655 and the *idi* gene of *D. wulumuqiensis* R12 | this study |
| pRSF-dxsispAR12ididxr | Kana^R^，pRSF-Duet-1 with the *dxs*、*ispA*、*dxr* genes of *E.coli* MG1655 and the *idi* gene of *D. wulumuqiensis* R12 | this study |
| *E. coli* DH5α | F-φ80 Lac ZΔM15 Δ(LacZYA-argF) U169 endA1 recA1 hsdR17(rk^-^,mk^+^) supE44λ- thi -1 gyrA96 reLA1 phoA | Vazyme |
| *E. coli* BL21(DE3) | F- ompT hsdS_B_(rB^-^ mB^-^ ) gaL dcm(DE3) | Vazyme |
| *E. coli* MG1655 | K-12 F^-^λ^-^ rph-1 | Vazyme |
| *D. wulumuqiensis* R12 | Aerobic, Gram-positive, non-spore-producing, nonmotile, tetracocci | Laboratory |
| H0 | *E. coli* BL21(DE_3_) strain containing pET-IEB | this study |
| H0E | *E. coli* BL21 (DE_3_) containing pET-IEB and pRSF-E | this study |
| H0B | *E. coli* BL21 (DE_3_) containing pET-IEB and pRSF-B | this study |
| H0I | *E. coli* BL21 (DE_3_) containing pET-IEB and pRSF-I | this study |
| H0dxs | *E. coli* BL21 (DE_3_) containing pET-IEB and pRSF-dxs | this study |
| H0dxr | *E. coli* BL21 (DE_3_) containing pET-IEB and pRSF-dxr | this study |
| H0idi | *E. coli* BL21 (DE_3_) containing pET-IEB and pRSF-idi | this study |
| H0ispA | *E. coli* BL21 (DE_3_) containing pET-IEB and pRSF- ispA | this study |
| H0R12dxs | *E. coli* BL21 (DE_3_) containing pET-IEB and pRSF-R12dxs | this study |
| H0R12dxr | *E. coli* BL21 (DE_3_) containing pET-IEB and pRSF-R12dxr | this study |
| H0R12idi | *E. coli* BL21 (DE_3_) containing pET-IEB and pRSF-R12idi | this study |
| H0R12ispA | *E. coli* BL21 (DE_3_) containing pET-IEB and pRSF-R12 ispA | this study |
| H21 | *E. coli* BL21 (DE_3_) containing pET-IEB and pRSF-dxsR12idi | this study |
| H22 | *E. coli* BL21 (DE3) containingpET-IEB and pRSF-dxsispA | this study |
| H23 | *E. coli* BL21 (DE3) containingpET-IEB and pRSF-R12ididxr | this study |
| H31 | *E. coli* BL21 (DE3) containingpET-IEB and pRSF-dxsispAR12idi | this study |
| H32 | *E. coli* BL21 (DE3) containingpET-IEB and pRSF-dxsR12ididxr | this study |
| H4 | *E. coli* BL21 (DE3) containingpET-IEB and pRSF-dxsispAR12ididxr | this study |

Table S2 Primers used in this work

| Primer | Sequences (5’-3’) | Note |
| --- | --- | --- |
| E-F | TATCATATGATGCGTCCCGAACTG | *Nde*I |
| E-R | ATGTATATCCTCCTTATCACTTCTCCCGCG | SD+AS |
| B-F | TAAGGAGGATATACATGTGACGGAATTTTCGC | SD+AS |
| B-R | AATCTCGAGTCAGCCGTGGGC | *Xho*I |
| I-F | TATGGATCCGATGACATCCCCTC | *Bam*HI |
| I-R | TTAGAATTCTCAGCGCCGGATGTC | *Eco*RI |
| pET-F1 | ATGCGTCCGGCGTAGA | */* |
| pET-R1 | GATTATGCGGCCGTGTACAA | */* |
| pET-F2 | TTGTACACGGCCGCATAATC | */* |
| pET-R2 | GCTAGTTATTGCTCAGCGG | */* |
| dxs-F | GCCGGATCCGATGAGTTTTGATATTG | *Bam*HⅠ |
| dxs-R | TTAGAATTCTTATGCCAGCCAGGC | *Eco*RⅠ |
| dxr-F | GTTGGATCCGATGAAGCAACTCACCATT | *Bam*HⅠ |
| dxr-R | ATTAAGCTTTCAGCTTGCGAGACGC | *Hin*dⅢ |
| idi-F | TTTGGATCCGATGCAAACGGAACACGT | *Bam*HⅠ |
| idi-R | CCGAAGCTTTTATTTAAGCTGGGTAAATGCA | *Hin*dⅢ |
| ispA-F | AATGGATCCGATGGACTTTCCGCAGCA | *Bam*HⅠ |
| ispA-R | GCCAAGCTTTTATTTATTACGCTGGATGATGTAG | *Hin*dⅢ |
| R12dxs-F | TATGGATCCGATGAACGAACTTCCCGG | *Bam*HⅠ |
| R12dxs-R | ATTAAGCTTCTACACCTCAATCGGCACG | *Hin*dⅢ |
| R12dxr-F | ATTGGATCCGATGTCGGGCGTGAG | *Bam*HⅠ |
| R12dxr-R | ATTAAGCTTTCATGCCCGCACCCC | *Hin*dⅢ |
| dxs-R1 | ATGTATATCCTCCTTATTATGCCAGCCAGGC | SD+AS |
| ispA-F1 | TAAGGAGGATATACATATGGACTTTCCGCAGCA | SD+AS |
| ispA-R1 | GCCGAATTCTTATTTATTACGCTGGATG | *Eco*RⅠ |
| R12idi-F | TATCATATGAGCCGCATCGAAGCGCGCAA | *Nde*I |
| R12idi-R | TAACTCGAGTGTATATCCTCCTTATCAACCCGGCTTTCGCAG | *Xho*I  SD+AS |
| dxr-F1 | TAAGGAGGATATACAATGAAGCAACTCACCATT | SD+AS |
| dxr-R1 | GGTTTCTTTACCAGACTCGAGTCAGCTTGCGAGACGC | *Xho*I |
| ispA-R2 | CGCGAGCTCTTATTTATTACGCTGGAT | *Sac*I |
| idnTF | ACCACCGGAAGGCTTATT | / |
| idnTR | GCAGGGTTACCGACAAAT | / |
| dxsF | CGGCAGAAAAAGACCCAA | / |
| dxsR | ACAACCAGTCGCCAAAGA | / |
| dxrF | AGCATAATCTGGGATACG | / |
| dxrR | GAAATTTTACGCCCCATC | / |
| idiF | CACACGGCAGACACCCTC | / |
| idiR | TCGTTGCTTTCTCCCAGT | / |
| ispAF | GCTGGCGACGCTTTACAA | / |
| ispAR | GCCTGACCACCGCACATT | / |
| crtBF | TGACGGAATTTTCGCCTGCC | / |
| crtBR | TCAGGTCCCGGCAAAACTG | / |
| crtEF | TCCTGACCATGATTCACCGC | / |
| crtER | CGGAACGATGACCGTGTAGT | / |
| crtIF | TTTTCCTTCGAGACGCTGCT | / |
| crtIR | CGTAGTGAATGCCCCAGGTT | / |

Table S3 Combination of *dxs*, *dxr*, *idi* and *ispA* genes in MEP pathway

| Gene/Strain  Combinatorial | *dxs* | *dxr* | R12*idi* | *ispA* | Strain |
| --- | --- | --- | --- | --- | --- |
| Double gene combination | + |  | + |  | H21 |
|  | + |  |  | + | H22 |
|  |  | + | + |  | H23 |
| Triple gene combination | + | + | + |  | H31 |
|  | + |  | + | + | H32 |
| Quadruple gene combination | + | + | + | + | H4 |

a


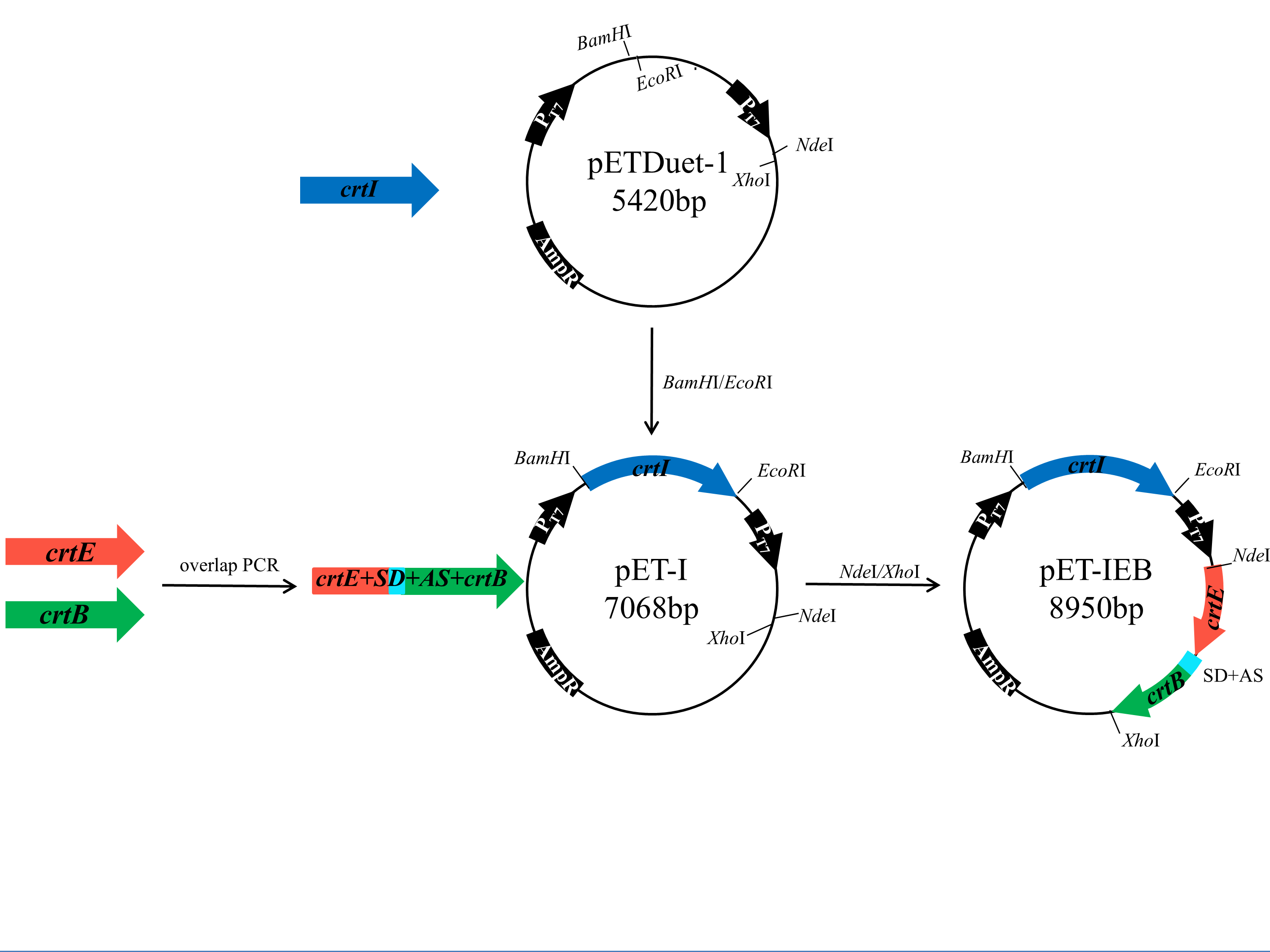


b


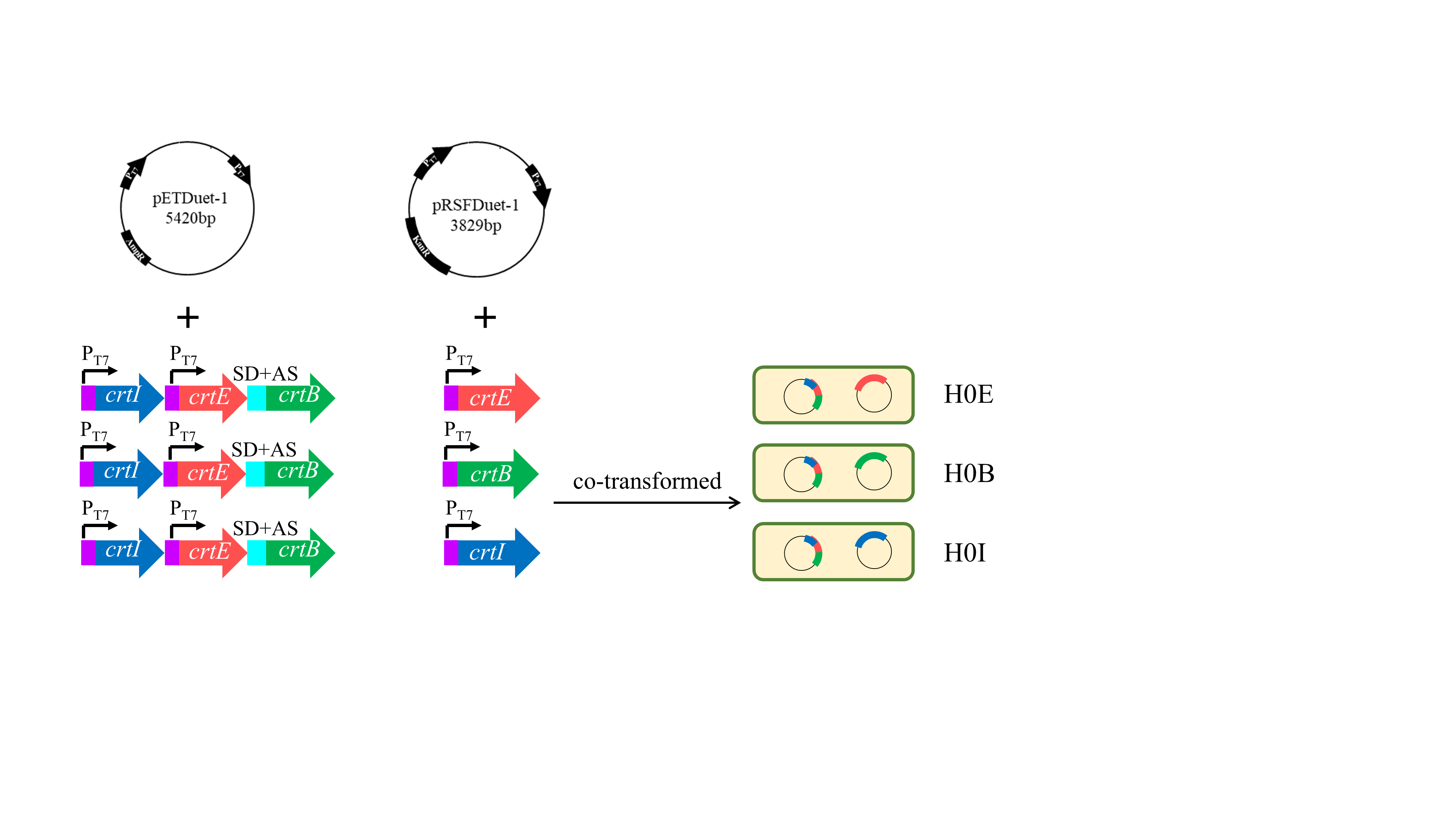


c


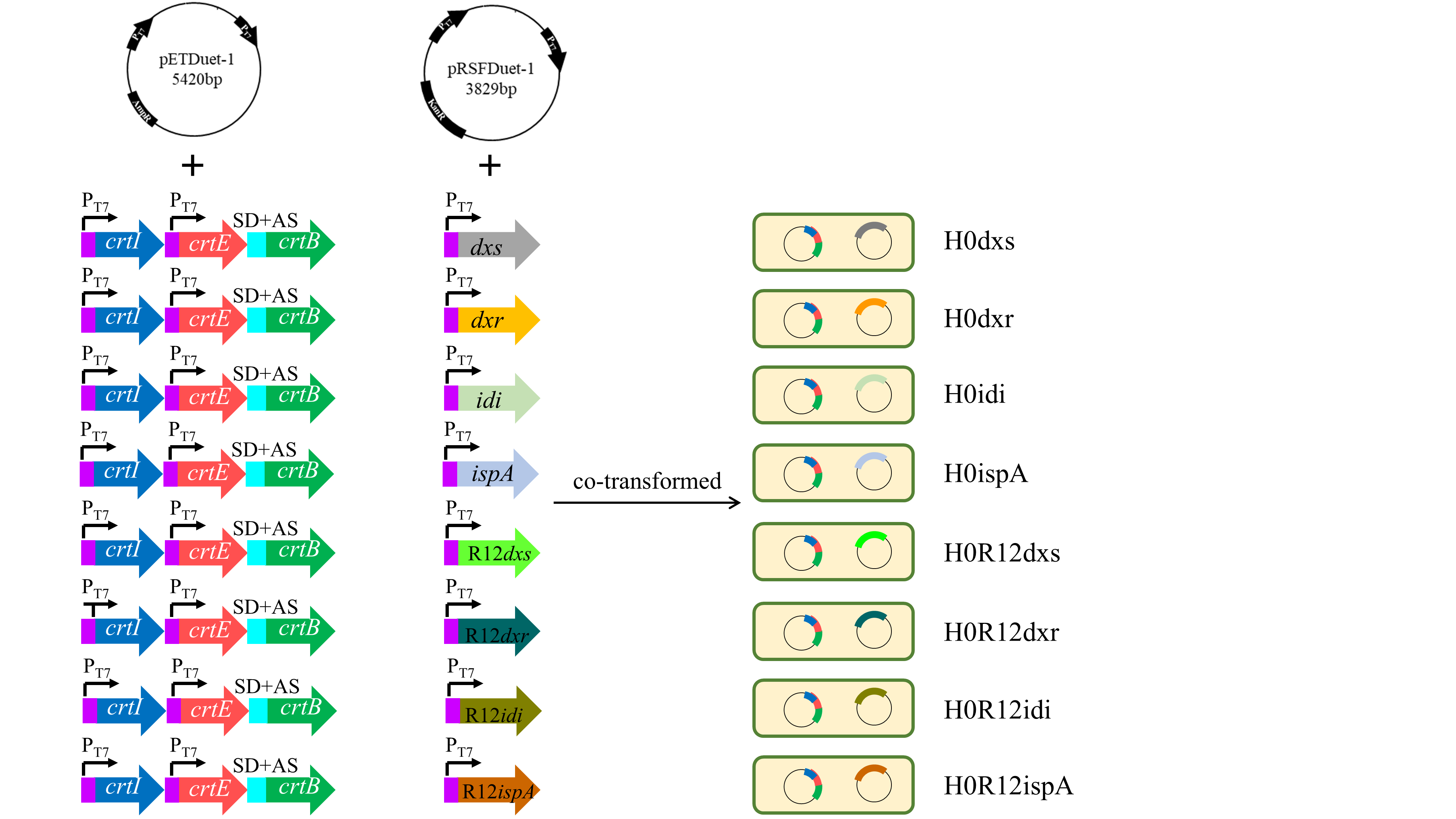


d


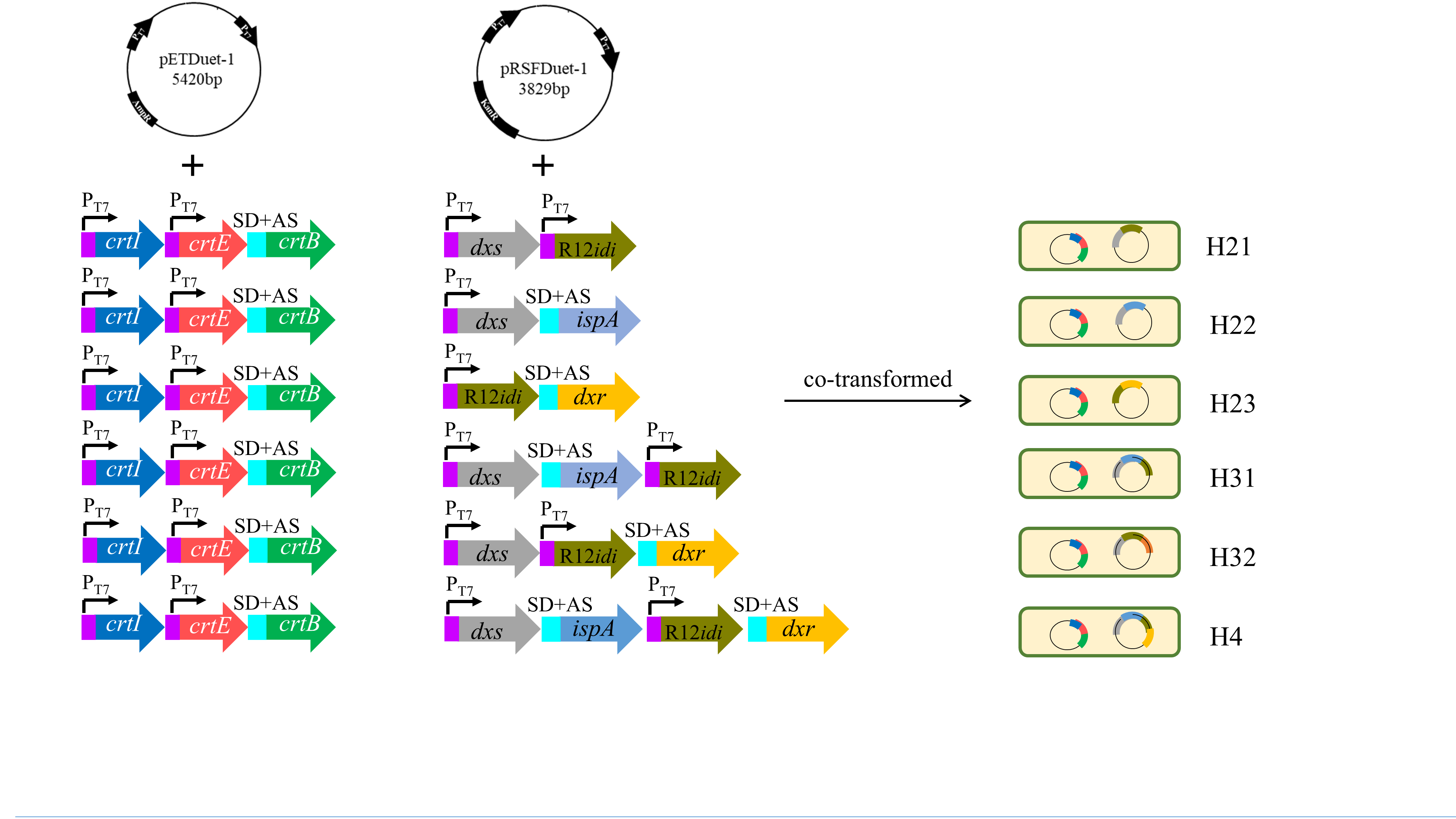


Figure S1 Construction strategies of lycopene synthesis pathway and MEP pathway in engineered *Escherichia coli*

(a) Construction of recombinant plasmid forthree key enzymes in the lycopene synthesis pathway; (b) Construction of multi-copy recombinant plasmids and recombinant strains of three key enzymes of the lycopene synthesis pathway; (c) Construction of recombinant plasmids and recombinant strains for single gene overexpression of key enzymes of the MEP pathway; (d) Construction of recombinant plasmids and recombinant strains overexpressing key enzymes of the MEP pathway in multigene combinations

Figure S2 Lycopene production and cell density of strain H0

b

d

a

c

e

Figure S3 Optimization of medium composition for lycopene synthesis

(a) Effect of carbon source on lycopene titer and cell growth; (b) Effect of sodium pyruvate concentration on lycopene titer and cell growth; (c) Effect of nitrogen source on lycopene titer and cell growth; (d) Effect of inoculation amount on lycopene titer and cell growth; (e) Dissolved oxygen concentration at different inoculum amount

a

b

c

Figure S4 Optimization of external culture conditions for lycopene synthesis

(a) Effect of cell growth time on lycopene titer and cell growth; (b) Effect of lactose concentration on lycopene titer and cell growth; (c) Effect of induction temperature on lycopene titer and cell growth.

Figure S5 Cell density and lycopene yield of the strain H21 over time during shake flask culture

Figure S6 Lycopene titer and plasmid stability of strain H21 after 10 rounds of subcultivation
